# Knowledge about breast cancer and reasons for late presentation by cancer patients seen at Princess Marina Hospital, Gaborone, Botswana

**DOI:** 10.4102/phcfm.v5i1.465

**Published:** 2013-10-02

**Authors:** Deogratias Mbuka-Ongona, John M. Tumbo

**Affiliations:** 1 Department of Family Medicine & Primary Health Care, University of Limpopo (Medunsa Campus), South Africa

## Abstract

**Introduction:**

In Botswana, breast cancer, the second most common malignancy amongst women, is often diagnosed late, with 90% of patients presenting at advanced stages at Princess Marina Hospital (PMH) Gaborone, the only referral hospital with an operational oncology department. The reasons for this late presentation have not been studied. Determination of these reasons is critical for the formulation of strategies to reduce morbidity and mortality from breast cancer in Botswana. The aim of this study was to explore existing knowledge about breast cancer and the reasons for late presentation amongst patients attending the oncology unit of Princess Marina Hospital.

**Method:**

A descriptive qualitative study using free attitude interview was performed. Twelve breast cancer sufferers were purposefully selected and eleven interviews conducted. Interviews were audio-taped, transcribed verbatim and translated. Thematic analysis of data was performed.

**Results:**

This study found that breast cancer sufferers had had poor knowledge of the disease prior to the diagnosis. Their knowledge improved markedly during their attendance to the oncology clinic. Screening methods such as breast self-examination (BSE) were not used frequently. The majority of participants had delayed going to the hospital because of a lack of knowledge, fear of the diagnosis and fear of death, misinterpretation of the signs, the influence of lay beliefs and advice from the community. In some cases, however, advice from family and friends resulted in a timely medical consultation. The poor clinical practices of some health workers and the inadequate involvement by decision makers regarding the issue of cancer awareness discouraged patients from seeking and adhering to appropriate therapy.

**Conclusions:**

Awareness and knowledge of breast cancer was found to be poor amongst sufferers prior to their diagnosis, but their awareness and knowledge improved after the diagnosis. There was limited use of screening methods and a generally delayed seeking of medical attention. The need for increased awareness and use of screening practices was identified to be essential for early diagnosis of the disease and for improved outcomes of breast cancer management in Botswana.

## Abstrait

**Connaissances sur le cancer du sein et les raisons de la présentation tardive de patients atteints de cancer observés à l'hôpital Princess Marina, Gaborone, Botswana**

**Introduction:**

Au Botswana, le cancer du sein, la deuxième cause d'infections malignes chez les femmes, est souvent diagnostiqué tardivement, 90% des patientes se présentant à un stade avancé de la maladie à l'hôpital Princess Marina (PMH) de Gaborone, l'unique hôpital de référence doté d'un service d'oncologie. Les raisons de cette présentation tardive n'ont pas été étudiées. Il est essentiel que ces raisons soient déterminées afin de pouvoir formuler des stratégies destinées à réduire la morbidité et la mortalité associées au cancer du sein au Botswana. L'objectif de cette étude était d’étudier les connaissances actuelles sur le cancer du sein et les raisons de la présentation tardive des patientes se rendant au service d'oncologie de l'hôpital Princess Marina.

**Méthode:**

Une étude qualitative descriptive avec entretien non directif a été utilisée. Douze femmes souffrant d'un cancer du sein ont été volontairement sélectionnées et onze entretiens ont été réalisés. Les entretiens ont été enregistrés, transcrits mot à mot et traduits. Une analyse thématique des données a été réalisée.

**Résultats:**

Cette étude a révélé que les personnes souffrant de cancers du seins connaissaient mal la maladie avant qu'elle ne soit diagnostiquée. L’étendue de leurs connaissances s'est considérablement améliorée durant leur visite au service d'oncologie. Les méthodes de dépistage telles que l'autopalpation des seins n’étaient pas utilisées fréquemment. La majorité des participants ont retardé leur visite à l'hôpital en raison d'un manque de connaissances, de la peur du diagnostic et de la peur de la mort, d'une mauvaise interprétation des signes, de l'influence des croyances établies et des conseils fournis par la communauté. Cependant, dans certains cas, les conseils de la famille et des amis leur ont permis de consulter à temps. Les mauvaises pratiques cliniques de certains travailleurs de la santé et l'implication inadéquate des décideurs quant au problème de la sensibilisation au cancer ont découragé les patientes de rechercher une thérapie appropriée et de la suivre.

**Conclusions:**

D'après cette étude, la sensibilisation au cancer du sein et les connaissances sur la maladie font défaut chez les malades avant qu'elles ne soient diagnostiquées, mais leur sensibilisation et les connaissances s'améliorent une fois le diagnostic prononcé. Le recours aux méthodes de dépistage était limité et la recherche de soins médicaux était généralement retardée. Il est essentiel d'améliorer la sensibilisation et d'utiliser des pratiques de dépistage en vue d'un diagnostic précoce de la maladie ou de meilleurs résultats du traitement du cancer du sein au Botswana.

## Introduction

Globally, breast cancer (Ca Breast) is the cancer most frequently reported amongst women, with an estimated 1.38 million new cancer cases diagnosed in 2008 (23% of all cancers). It ranks second overall (10.9% of all cancers). It is the most common cancer both in developed and developing regions, with an estimated 690 000 new cases reported each year in each region (population ratio 1:4).^1^ Incidence rates vary from 19.3 per 100,000 women in Eastern Africa to 89.7 per 100,000 women in Western Europe. In 2006 the global risk of women developing breast cancer in their lifetime was estimated to be ‘1 in 8’.^2^

### Background

Breast cancer remains a major cause of morbidity and mortality in sub-Saharan Africa. In 2008 it had an incidence rate of 38/100 000 and mortality rate of 20/100 000 in that region.^1^ In Botswana breast cancer is the second most common malignant disease and its occurrence is on the increase. The Botswana national cancer registry, published in 2005, showed that between 1986 and 2004 Ca breast accounted for 26.3% of all recorded malignancies and was second only to cancer of the cervix (30.7%).^3^ According to the World Health Organisation (WHO), Botswana ranked number 170 in the world in 2011 in terms of deaths caused by breast cancer. Breast cancer deaths in Botswana reached 0.30% of total deaths; death rate was 9.09 per 100 000 of population.^4^

According to WHO the burden of cancer can be reduced by enhanced knowledge about the causes of cancer and interventions to prevent and manage the disease. Implementing evidence-based strategies for cancer prevention, early detection of cancer and management of patients with cancer can reduced its incidence.^5^ However, such strategies are not universally available and this may possibly contribute to the late presentation and high mortality in developing countries. In Botswana the disease is commonly diagnosed late, 90% of patients presenting at advanced stages at Princess Marina Hospital (PMH), Gaborone, the only referral hospital in Botswana with an operational oncology department.^3^ The reasons for this late presentation in patients with breast cancer had not been studied in Botswana prior to this study and were therefore not known when the study was carried out. Determining these reasons is vital for the formulation of intervention strategies to reduce morbidity and mortality from breast cancer in Botswana.

#### Aims of the study

The aim of this study was to explore the knowledge of breast cancer patients about their disease and to establish the reasons for their late presentation at the Princess Marina Hospital (PMH), Botswana.

#### Contribution to field

This study goes a long way in correcting the misconception that breast cancer is not common in the African communities. It also highlights the difficulties that communities from disadvantaged backgrounds face with regard to access to health services for breast cancer.

## Research methods and design

### Materials design

A qualitative study using free attitude interviews was conducted in 2007. The study population consisted of all patients diagnosed with breast cancer seen and managed at PMH in Gaborone, Botswana. This is the only referral hospital providing oncology services in the country. Twelve adult patients with breast cancer who consented to participate in the study were purposively selected. Only eleven were interviewed because one withdrew from the study before being interviewed. Patients under 18 years and those who were very ill or mentally incapacitated were excluded from the study.

### Procedures and analyses

Interviews were conducted in English or Setswana by a trained research assistant according to the participant's preference. The exploratory question was: ‘*Tell me everything that you know about breast cancer and how you have been caring for yourself with regard to the disease.’* Reflective summaries and clarification were undertaken during each interview by the interviewer. Interviews were audio-taped and field notes taken by the researcher for triangulation. Verbatim transcription and translation of the interviews were performed by a trained research associate. The ‘cut and paste’ method was used to identify themes, with supporting evidence in the form of quotations from the transcripts. Member checks were performed with the participants to verify the accuracy of the data.

## Results

### Profile of participants

All eleven participants were Black females with a mean age of 54 years; their ages ranged between 37 and 76. The majority of the participants (78%) were unmarried and unemployed and each had more than three children (see table 1). The period since their diagnosis with breast cancer ranged between one and nine years (mean duration 3 years 7 months) and the mean duration from the onset of symptoms to their seeking help at the hospital was 3 years, with a range between 1 and 10 years. The most common presentation of breast cancer amongst the participants was a painless lump, followed by bloody nipple discharge. The majority were classified as stage three of the disease (cancer has spread to axillary lymph nodes, which are clumped together or attached to other structures, and/or it extends to the chest wall and/or skin of the breast).

**TABLE 1:** Characteristics of participants.

Participant	Sex	Age	Marital status	Number of children	Highest education level
P1	Female	43	Unmarried	3	Primary school
P2	Female	61	unmarried	3	tertiary
P3	Female	45	Married	3	tertiary
P4	Female	45	Unmarried	1	No schooling
P5	Female	37	Unmarried	6	Secondary school
P6	Female	52	Unmarried	7	No schooling
P7	Female	76	Unmarried	7	No schooling
P8	Female	38	Unmarried	0	Tertiary
P9	Female	56	Married	6	Primary school
P10	Female	57	unmarried	3	No schooling
P11	Female	31	married	1	Secondary school

#### Emerging themes

Themes emerging from the interviews included an understanding of risk factors for developing breast cancer, hospital attendance, diagnosis of breast cancer, patients’ knowledge, information and misconception about breast cancer, delay in seeking help, poor screening activities, health practitioners’ laxity, the influence of traditional healers, emphasis on Human immunodeficiency virus (HIV) and limited access to care in rural areas (see table 2).

**TABLE 2:** Summary of emerging themes.

Number	Theme	Illustrative quote
1	Risk factors for developing breast cancer	‘… now I think this is the same thing which is affecting me because these people are my relatives, how could I escape …’
2	Hospital attendance and diagnosis of breast cancer improved patients’ knowledge	‘… when I came to Princess Marina Hospital, I realized that many women suffered from this disease and were not aware …’
3	Information about breast cancer readily available	‘… in fact some of this I read …’ ‘Sometimes you listen from the radio …’
4	Awareness of symptoms motivate patients to seek help	‘… I told my relatives that I have a lump in the breast … they advised me to go to the hospital so that lump could be removed …’
5	Delay seeking help at hospital because of poor knowledge about breast cancer	‘The fact that the lump is painless usually makes people to delay without seeking help from the hospital …’
6	Delay going to the hospital because of fear of the diagnosis and death	‘… if you have something on the breast, they never want to talk about it because they are afraid they are going to be told it is cancer and when they are told that it is cancer they know they are going to die …’
7	Misconception and misinformation about breast cancer	‘And from the people saying if you have cancer, you will die …’
8	Health practitioners’ lack of seriousness about breast cancer	‘… I just kept on asking doctors, they always ignored me, they did not even write on my outpatient card …’ ‘… it took about two and half years before they did a biopsy …’
9	Influence of traditional healers	‘… a traditional doctor tells you stories, like this other lady last time who said she had three years struggling with cancer. She had been going to traditional doctors and was told it was witchcraft.’
10	Infrequent screening activities for breast cancer	‘Women are advised to check themselves because cancer of the breast is increasing, widespread and common but they don't.’
11	Human immunodeficiency virus (HIV) overshadows breast cancer	‘… and it seems people are much concerned about HIV and cancer awareness they are not much concerned about it …’
12	Disadvantaged remote rural areas	‘… this awareness walk, it seems to be just in Gaborone and in some other places it is not being done; if only a lot was being done it would really help people …’

1. *Risk factors for developing breast cancer:* Heredity, contraceptive use and the reproductive cycle were correctly identified as important risk factors for the development of breast cancer:
  ‘Cancer of the breast can be hereditary, if you had a relative who suffered from breast cancer …’ (P11, Female, 31)
  ‘… I had three aunties who were affected by cancer of the breast … now I think this is the same thing which is affecting me because these people are my relatives, how could I escape …’ (P10, female, 57)
  ‘… or when you start menstruation at an early age …’ (P11, Female, 31)
  ‘… I thought contraceptives can affect the breast …’ (P6, Female, 52)
2. *Prior knowledge:* Knowledge and awareness regarding breast cancer was poor prior to diagnosis but improved during hospital attendance:
  ‘… I really not heard anything about this type of cancer; the only types I ever heard of were the cancer of oesophagus, cervical cancer as well as cancer of the bone …’ (P5, Female, 37)
  ‘Before I thought that for one to have Ca breast you must have a lump not a discharge, it seems it is not like that, you can have a lump, you might not have a lump but still can still have cancer of the breast without a lump.’ (P2, Female, 61)
  ‘… when I came to Princess Marina Hospital, I realized that many women suffered from this disease and were not aware …’ (P5, Female, 37)
  ‘... I realized that I had a small lump in my breast, I wondered what the lump was for …’ (P6, female, 52)
  ‘… if they feel a lump in the breast they should know that it is breast cancer …’ (P2, Female, 61)
  ‘You can either have a lump or some fluid coming from your breast … sometimes breast cracks and bleeds …’ (P4, Female, 45)
3. *Information about breast cancer readily available:* In Botswana, information about breast cancer was readily available and accessible. Information can be gained from the media, printed materials, health facilities and community members:
  ‘… I heard from people as we were discussing … and also from the hospital teachings …’ (P1, Female, 43)
  ‘… in fact some of this I read …’ (P2, Female, 61)
  ‘Sometimes you listen from the radio …’ (P2, Female, 61)
  ‘… I heard over the radio …’ (P2, Female, 61)
  ‘… I decided that they would remove the whole breast because I always hear from the radio that the remaining part of the breast could be dangerous …’ (P11, Female, 31)
4. *Awareness of symptoms motivate patients to seek help:* Enhanced awareness regarding symptoms of breast cancer and advice from relatives and health professionals encouraged participants to seek help early:
  ‘… I told my relatives that I have a lump in the breast … they advised me to go to the hospital so that lump could be removed …’ (P11, Female, 31)
  ‘… I then decided to see the doctors immediately because I had already heard about breast cancer …’ ( P10, Female, 57)
  ‘When lumps develop or a wound on the breast you should run to the hospital.’ (P9, Female, 56)
  ‘You have to do what is called breast self-examination … you palpate your breast to check the lumps, if you find any lump you need to go and see a doctor …’ (P2, Female, 61)
  ‘When things develop in the breast you should run to the hospital, even when you develop a wound or lump you should go to see the doctor to find out what is wrong with you …’ (P4, Female, 45)
5. *Delay in seeking help at the hospital because of a lack of knowledge about breast cancer:* The majority of participants delayed going to the hospital because of the lack of awareness and knowledge regarding breast cancer:
  ‘The fact that the lump is painless usually makes people to delay without seeking help from the hospital …’ (P3, Female, 45)
  ‘Before I thought that for one to have Ca breast you must have a lump not a discharge, it seems it is not like that, you can have a lump, you might not have a lump but still can still have cancer of the breast without a lump.’ (P2, Female, 61)
  ‘I had a lump … because you don't feel any pain … I should think that is the reason why most of the time many patients are diagnosed at an advanced stage …’ (P8, Female, 61)
6. *Delays in going to the hospital because of a fear of the diagnosis and death*:
  ‘It was big; I told the doctor that I didn't want to go to Nyangabwe referral hospital (NRH) because they would remove my breast …’ (P1, Female, 43)
  ‘She [*my mother*] asked me if I want to go to the hospital so that my breast will be removed and I die …’ (P1, Female, 43)
  ‘… if you have something on the breast, they never want to talk about it because they are afraid they are going to be told it is cancer and when they are told that it is cancer they know they are going to die …’ (P2, Female, 61)
7. *Misconception and misinformation about breast cancer*: Misinformation and misconceptions about breast cancer amongst community members were common reasons for delays in seeking help:
  ‘… And from the people saying if you have cancer, you will die …’ (P11, Female, 31)
  ‘… the advices were mixed, some were saying go for operation, some were saying no you should not go for operation …’ (P3, Female, 45)
  ‘… one of my friends wanted me to have alternative medication …’ (P2, Female, 61)
  ‘Some people said [*if*] they could have known before I had surgery they could have helped me with some herbs to treat the cancer. This would not have helped.’ (P2, Female, 61)
  ‘I thought it was simple; after doing those treatment it will be complete but what I realized is that you can do radiotherapy and chemotherapy and you can still remain like that and you can do it the second time, the third time, because one of my friends did it first time second time third time and she was gone …’ (P8, female, 38)
  ‘… because I have many children maybe breast cancer can be caused by breastfeeding …’(P6, Female, 52)
8. *Health practitioners’ lack of seriousness about breast cancer*: Participants expressed disappointment and annoyance at the lack of seriousness in some health practitioners in facilities other than PMH regarding breast cancer:
  ‘… doctors sometimes, they take matters easy like the Nyangabwe doctor, he was just so taking it easy …’ (P3, Female, 45)
  ‘… I just kept on asking doctors, they always ignored me, they did not even write on my outpatient card …’ (P9, Female, 56)
  ‘… it took about two and half years before they did a biopsy …’ (P2, Female, 61)
  ‘… agree now he was forgetting the lump ah I got annoyed, I went to a private doctor …’ (P3, Female, 45)
  ‘… showing me that when you do this operation you should know your status and all that … not just saying know your status you see … they should learn to talk to different patients … they should know that one is traumatized already …’(P8, Female, 38)
  ‘… they said ok, if you don't undergo this operation we are just going to give you chemotherapy and radiotherapy, maybe you may survive for two and half months, then you will be gone. Ah you know that was so scaring …’ (P8, Female, 38)
  ‘… they just tell us no this is your condition and we just have to do this and when you ask is there any alternative way of treating this condition, they will say no, no, no, no, it is too late you have to do like this, you have to do like this …’ (P8, Female, 38)
  ‘… the approach was not good; when I went to the private doctors they also said something about HIV and breast cancer but it was in a nice manner …’ (P3, Female, 45)
9. *Influence of traditional healers*: Participants were quite aware of the ineffectiveness of spiritual and traditional treatment in the management of breast cancer. They reported that that kind of treatment aggravated the condition because of the traditional and spiritual healers’ lack of expertise:
  ‘… a traditional doctor tells you stories, like this other lady last time who said she had three years struggling with cancer. She had been going to traditional doctors and was told it was witchcraft. Then she was eventually advised by someone to go to the hospital, she didn't last long, she was buried … so I just thought if I can go to the traditional doctor, I am going to end up dead …’ (P9, Female, 56)
  ‘Traditional healing is not good because their herbs would not cure the disease … after a while the disease would be severe …’ (P11, Female, 31)
  ‘… they do not have the expertise that the medical doctors have … cannot perform X-rays to see what the patient is suffering from … the medical doctors do tests and X-rays to diagnose what the patient is suffering from …’ (P5, Female, 37)
  ‘I wanted to see the traditional healers but then I decided to go to the hospital …’ (P4, Female, 45)
10. *Infrequent screening activities for breast cancer*: Screening activities such as breast self-examination (BSE) were infrequently performed despite advice:
  ‘They checked the breast and they said you should always be checking for lump. So I kept on doing that but not that frequently …’ (P11, Female, 31)
  ‘Women are advised to check themselves because cancer of the breast is increasing, widespread and common but they don't.’ (P1, Female, 43)
  ‘You have to do what is called breast self-examination … you palpate your breast to check the lumps, if you find any lump you need to go and see a doctor …’ (P2, Female, 61)
11. *Human immunodeficiency virus (HIV) overshadows breast cancer*: Participants noted that decision makers were more concerned about HIV than about breast cancer:
  ‘… and it seems people are much concerned about HIV and cancer awareness, they are not much concerned about it …’ (P3, Female, 45)
  ‘… he said ah, do you know your status? What status, HIV? I said, I did test when I was doing head surgery in 2002 and it was negative. He said no, no, no, go to room eleven and check your status for HIV and stop thinking about cancer of breast …’ (P3, Female, 45)
  ‘… many people are affected by cancer, not only breast cancer and it seems people are much more concern about HIV …’ (P8, Female, 38)
12. *Disadvantaged remote rural areas:*
  ‘… when I first developed lump in the breast I was in the cattle post and I took a very long time before going to the hospital … when I went to the hospital the lump had already grown too big …’ (P5, Female, 37)
  ‘… this awareness walk, it seems to be just in Gaborone and in some other places it is not being done; if only a lot was being done it would really help people …’ (P8, Female, 38)

##### Ethical consideration

Ethical approval for this study was obtained from the Research Ethics and Publications Committee of the University of Limpopo (Medunsa Campus), clearance certificate number: MP14/2006

## Discussion

This study found that female patients diagnosed with breast cancer and managed at the only public health oncology unit in Botswana had poor knowledge and awareness about the disease prior to their diagnosis, but that their knowledge improved after their diagnosis. Knowledge was obtained from a variety of sources, including the media and health care providers. The study did not seek to find out at what point in time relative to the date of diagnosis the participants gained their knowledge as information about breast cancer is readily available in Botswana. The purposive selection of participants who had already been diagnosed with breast cancer and given much information about the disease can explain why the findings of this study are different from those of studies that depicted a lack of knowledge in a rural community in Nigeria.^6^ This study was comparable to other studies conducted in Nigeria and South Africa in which only a few participants were knowledgeable about the risk factors and the role of heredity in breast cancer.^6, 7, 8^ The awareness of risk factors and the role of heredity should be disseminated in the community. Risk factors related to dietary habits and a combination of menopause and being overweight were not reported by any of the participants in the study, and these should also be also brought to the attention of the population at risk of the disease in the form of a community oriented health promotion.^9, 10^

Reasons for not seeking help immediately included fear, lay beliefs, poor advice, the negative influence of traditional healers, an over-emphasis on HIV, perceived poor communication by health professionals and their lack of interest in breast cancer. It was evident from this study that the general views of communities were a major determinant of help-seeking behaviour, and this finding agrees with the findings of a study carried out in Canada amongst Asian women that found that religion, culture and the views of the community determined help-seeking behaviour.^11, 24^

It was also evident that screening methods for breast cancer were infrequently used. Late presentation of breast cancer is not a new trend globally, and it has been described, for example, in the sub-Saharan region. A study from the University of Benin, Nigeria reported a high proportion of women (78%) who presented at stages three and four of breast cancer.^12, 13^ In South Africa about 77% of black women presented at stages three and four of the disease, according to the national cancer registry covering the period of 1986 to 1992.^14^

There are some similarities and differences in the reasons for late presentation of the disease between countries with different levels of development. In the United States of America reasons include a lack of education and knowledge about symptoms, risk factors and the benefits of early detection of breast cancer.^15^ In Africa ignorance, the use of alternative medicine and a fear of surgery were common reasons given for late presentation.^16^ The disease explanatory model based on cultural beliefs was an important determinant of the help-seeking behaviour in Africa. In many societies, witchcraft is perceived as a cause of cancer and this leads to delayed presentation at hospitals.^14^ Because of a lack of knowledge about breast cancer, a painless lump is not associated with the disease amongst rural women in some African countries.^19^ In the United Arab Emirates it was found that advancing age, low socioeconomic status, fear of the diagnosis, fear of the consequences of cancer treatment, misconceptions about the aetiology, denial and spirituality, including faith, were responsible for late presentation.^17^

Early detection of breast cancer is dependent on awareness and knowledge of screening techniques. Rural women have been reported to lack appropriate information about breast cancer and consequently also about early detection measures.^18^ In a study undertaken in Nigeria, women living in rural areas were found to have an extremely low level of awareness of breast cancer with minimal skills regarding breast self-examination and clinical breast examination.^19^ Another study conducted amongst South East Asian women living in Canada found that religion and culture were paramount reasons for women not to adhering to screening programmes.^24^

In developed countries increased awareness and knowledge do not necessarily result in enhanced use of screening procedures. In a survey performed in Austria, only 31% of the participants undertook breast self-examination (BSE), although 92% of them were aware of the practice.^20^ Similar gaps between knowledge and practice were shown amongst women in the United Arab Emirates, Iran and Australia and amongst Japanese American women.^17, 21, 22 ,23^

The majority of the participants in this study were unemployed and had not participated in screening activities. This is in agreement with the findings of a study amongst United Arab Emirates women which indicated that being employed proved to be an independent predictor for participation in three screenings examinations: breast self-examination (BSE), clinical breast examination (CBE) and mammography.^17^

## Limitations of the study

This study had the following limitations: The use of a qualitative design dictates that these findings cannot be generalised. The purposive sampling provided information unique to the participants who had experienced the disease and failed to depict the experiences of those not yet diagnosed with breast cancer. Insight into the practice and knowledge of those not suffering from breast cancer would be critical in the design of prevention and therapeutic strategies. As purposive sampling was used in this qualitative study, the findings are unique to the setting and cannot be generalised.

### Recommendations

1. In view of massive misconceptions about breast cancer, cancer awareness should be improved through media, health facilities on a regular scheduled activities to clear misconceptions in the communities
2. Breast self examination should be encouraged to identify breast cancer early
3. training in communication skills for health professional be considered especially for those dealing with cancer patient in this era of HIV where all effort seem to be deviated to the fight against HIV

## Conclusions

The findings of this study showed that amongst patients with breast cancer seen at the referral hospital in Botswana, knowledge of the disease was poor prior to diagnosis, but improved markedly after diagnosis and upon attending the hospital. Screening for breast cancer was infrequently performed. Patients with breast cancer generally delayed seeking help at the hospitals because of their fears, misinformation, misinterpretation of signs and cultural influences.
